# Variation in Malaria Transmission Dynamics in Three Different Sites in Western Kenya

**DOI:** 10.1155/2012/912408

**Published:** 2012-09-03

**Authors:** S. S. Imbahale, W. R. Mukabana, B. Orindi, A. K. Githeko, W. Takken

**Affiliations:** ^1^Laboratory of Entomology, Wageningen University, P.O. Box 8031, 6700 EH Wageningen, The Netherlands; ^2^Kenya Medical Research Institute, Centre for Global Health Research, P.O. Box 1578, Kisumu 40100, Kenya; ^3^School of Applied Sciences and Technology, Kenya Polytechnic University College, P.O. Box 52428-00200, Nairobi, Kenya; ^4^International Centre of Insect Physiology and Ecology, P.O. Box 30772-00100, Nairobi, Kenya; ^5^School of Biological Sciences, University of Nairobi, P.O. Box 30197-00100, Nairobi, Kenya

## Abstract

The main objective was to investigate malaria transmission dynamics in three different sites, two highland villages (Fort Ternan and Lunyerere) and a lowland peri-urban area (Nyalenda) of Kisumu city. Adult mosquitoes were collected using PSC and CDC light trap while malaria parasite incidence data was collected from a cohort of children on monthly basis. Rainfall, humidity and temperature data were collected by automated weather stations. Negative binomial and Poisson generalized additive models were used to examine the risk of being infected, as well as the association with the weather variables. *Anopheles gambiae s.s.* was most abundant in Lunyerere, *An. arabiensis* in Nyalenda and *An. funestus* in Fort Ternan. The CDC light traps caught a higher proportion of mosquitoes (52.3%) than PSC (47.7%), although not significantly different (*P* = 0.689). The EIR's were 0, 61.79 and 6.91 bites/person/year for Fort Ternan, Lunyerere and Nyalenda. Site, month and core body temperature were all associated with the risk of having malaria parasites (*P* < 0.0001). Rainfall was found to be significantly associated with the occurrence of *P. falciparum* malaria parasites, but not relative humidity and air temperature. The presence of malaria parasite-infected children in all the study sites provides evidence of local malaria transmission.

## 1. Introduction

There are large among-site variations in the abundance and temporal dynamics of malaria vector populations indicating that the risk of parasite transmission differs among sites [1]. Even in one topographic area, mosquito vectors and malaria infections may not be distributed homogeneously, and some households within the same area have a higher malaria incidence than others [2–4]. Many factors may be responsible for this spatial heterogeneity of malaria vectors and transmission intensity such as land use and land cover changes, topography, house building materials, and design and the level of household protection measures against mosquitoes [5–10]. In most cases, it is difficult to identify the factor that contributes most to these variations. In many African highlands, malaria resurgence has been attributed largely to the rise in drug-resistant parasites [11], although other factors are also likely to be important, such as poor health systems [12], land use such as deforestation and swamp reclamation [6, 13, 14], population growth and migration [15], and climate variability [16, 17].

In Western Kenya, malaria is predominantly a rural disease, and the main malaria vectors are *Anopheles gambiae sensu stricto, An. Arabiensis,* and *An. funestus* [18]. *Anopheles gambiae* generally increases in density after the start of the long rains, while *An. funestus* density is seen to vary in direct proportion to the proximity of permanent breeding grounds rather than rainfall [19]. In the adult stage, these anopheline species share many of the same habitats. In the Usambara Mountains, Tanzania, and in Western Kenya, Balls et al. [8] and Githeko et al. [5] reported that altitude plays an important role in determining malaria infection due to its effect on temperature. Temperature decreases with increasing altitude, and at lower altitudes, the high temperature levels accelerate the sporogonic cycle of malaria parasites in the presence of vectors and the breeding habitats. Land use such as deforestation and swamp reclamation by eliminating shade modifies the local climate and microclimate, and in the presence of stagnant water, new habitats for malaria vectors are formed [6, 14]. Consequently, the new habitats provide new breeding grounds leading to increased vector densities and subsequently an increase in malaria transmission. Over the past four decades, deforestation and swamp cultivation have widely occurred in Western Kenya, and these are now thought to be a major contributing factor to the abundance of breeding habits and the survival of malaria vectors. The ever-increasing human population and the need for food security place large pressure on land and threaten the survival of undisturbed natural forests and swamps. The current study was undertaken to investigate the dynamics of malaria transmission in three different sites in Western Kenya. The hypothesis being tested is that malaria risk is high in transformed swamp sites of Nyalenda and Lunyerere and not Fort Ternan.

## 2. Materials and Methods

### 2.1. Study Area

The study was carried out in Western Kenya in two highland villages, Lunyerere and Fort Ternan, and the lowland periurban Nyalenda, a suburb of Kisumu city. Fort Ternan (0° 12′ S and 35° 20′ E) is a rural village in Kericho County located on the slopes of Nandi hills lying between 1480 and 1650 m. The area is hilly with sharp, V-shaped valleys with high rainfall favouring agriculture. Farming in Fort Ternan is done on large scale with the main crops being sugarcane, maize, and to some extent coffee. Lunyerere (0° 06′ N and 34° 43′ E) village is located in Vihiga County, on the eastern side of the Kakamega forest, about 5 km north of the equator, with an altitude ranging from 1460 to 1550 m. The area is characterized by broad U-shaped valleys that are prone to flooding offering excellent mosquito breeding habitats. Majority of the valley bottoms in this area were previously forested covered with natural swamps that were fed by water through underground seepage. However, in recent times, the land has been cleared to farmland, where the community members practice small-scale food crop farming. Nyalenda (0° 06′ S and 34° 46′ E, 1100 m) is a periurban area located on the outskirts of Kisumu city. Kisumu is situated on the northeastern tip of Winam Gulf, an inlet of Lake Victoria. Nyalenda is fairly flat area fed by natural springs that produce abundant water used for irrigation on small-scale gardens. The area was previously a swamp but due to an increase in population in urban Kisumu, farming for food crops has been encouraged as a way of ensuring food security for the expanding population. More information of the study areas including the larval species and abundance can be found in Imbahale et al. [13]. Briefly, a study on the larval vector species composition found *An. arabiensis *to be the most abundant in Fort Ternan and Nyalenda, 71% and 93%, respectively, whereas *An. gambiae* s.s. was the most abundant vector species in Lunyerere (93%).

#### 2.1.1. Entomological Survey

Ten houses were randomly selected in Lunyerere and Nyalenda for adult mosquito sampling, while in Fort Ternan, 20 houses were randomly selected. Most of the sentinel houses consisted of mud walls and thatched roofs, while a few had iron sheet roofs and cemented walls. In each site, adult mosquitoes were collected monthly from the sentinel houses by Centres for Disease Control (CDC) battery-operated light traps (Model 512; John W. Hock Company, Gainesville, FL, USA) and pyrethrum spray catches (PSCs). Pyrethrum spray catch began in March 2006, while CDC light trap collections began later in July 2006. On each sampling occasion, the CDC light trap catches preceded the PSC catches by 24 h throughout the study. Light traps were installed in the sentinel houses near the foot end of the bed, next to an untreated bed net [20] and operated from 18.00 pm to 06.00 hours in each house. One day after the CDC light trap collections, PSCs were made between 08 : 00 and 11 : 00 am using simple flit guns to spray inside closed rooms with 2% pyrethrum extract synergised with piperonyl butoxide in kerosene [21]. Ten minutes were allowed before closed rooms were reentered, and the mosquitoes were collected from the sheets that had been laid out in the rooms. Female *Anopheles* mosquitoes were identified morphologically according to Gillies and Coetzee [22], stored, and dried on silica gel at room temperature pending further analysis. Although culicine mosquitoes do not transmit malaria, mosquitoes of this genus are mainly nuisance biters and were also recorded during the sampling. Mosquito sampling took place in the same sentinel houses throughout the study. In any event such as abandoning of the houses by occupants, an adjacent house replaced the original one. 

Members of the *An. gambiae *complex were identified to the species level using the polymerase chain reaction (PCR) method [23]; for this purpose, DNA of adult female *An. gambiae* were extracted from one wing or leg. The head and thorax of each female *An. gambiae *and* An. funestus* were tested singly for *Plasmodium falciparum* sporozoites using the standard enzyme-linked immunosorbent assay as described by Beier et al. [24] at the Walter Reed Army Institute Laboratory based at Kisian, Kisumu, Kenya. 

### 2.2. Parasitological Surveys

A house-to-house population survey was done in each study location to identify households with children aged between 2 and 10 years. These children were then enrolled to form a study cohort of 100 children per study site. In Fort Ternan, children were enrolled from houses located in the valley and up the valley. Consent was sought from the parents/guardians before the child was enrolled in the study. Each child was then given a unique code that was used to track the same child throughout the study from June 2006 to April 2008. During the surveys, the children were followed to their respective schools, while those not in schools were followed in their respective homes. Blood samples were collected monthly by the standard finger-prick method; thick and thin smears were prepared on labeled slides [25]. Core body temperature of the children was measured with a Braun Thermoscan (Frankfurt, Germany) ear probe thermometer, and each child was tested with a fresh sterile ear plug. The thin and thick blood smears were air dried. Thereafter, the thin and thick smears were fixed in methanol and stained in 4% Giemsa for 30 minutes. An experienced technician examined the slides under 1,000 magnification by using oil immersion to identify and count the parasite species. Random checks were carried out on the slide counts (to include at least 10% of all slides) by independent microscopists to ensure quality control. Parasite density was scored against 200 leukocytes when the slide was positive; otherwise, the whole slide was carefully scanned before being declared negative. An individual was considered positive if malaria parasites were detected in the blood smear. Any child that was clinically ill at the survey date was taken to the nearest public health facility for treatment free of charge. A child was considered clinically ill if he/she had fever (a core body temperature ≥ 37.5°C) and malaria parasites identified from the blood smear. Malaria parasite incidence studies in Fort Ternan commenced in June 2006 while in Lunyerere and Nyalenda in January 2007. 

### 2.3. Weather Data

Automatic weather stations were installed, one at the Fort Ternan Health centre, the other one at Lyanaginga Health Centre about 30 km from Lunyerere, and another at the Kenya Medical Research Institute (KEMRI), Centre for Global Research, Kisian, about 17 km from Nyalenda (but at the same altitude as Nyalenda). The weather stations measured temperature and humidity at 2 m above ground (ventilated probe; Vaisala, Finland) and precipitation (rain gauge, Eijkelkamp, The Netherlands) throughout the study period. The weather variables were recorded on a 21x Microdatalogger (Campbell Scientific Inc., UK) at an interval of 15 minutes from March 2006 to April 2008. Detailed description of how the weather station works has been provided in Paaijmans et al. [26]. For Lunyerere and Fort Ternan, all variables were measured as expected. In Kisian, however, the weather station experienced technical problems for several months; hence, humidity data are not available for Nyalenda. As a proxy, we have used the average relative humidity data from the Kenya Airports Authority based at Kisumu airport, midway between Nyalenda and Kisian.

### 2.4. Ethical Considerations

Institutional ethical clearance was given by the Kenya Medical Research Institute (KEMRI) and Wageningen University and Research Centre (WUR), The Netherlands, protocol approval numbers 1121 and 512. In addition, consent was obtained from the parents/guardians, community elders, and house owners. 

### 2.5. Data Analysis

The analyses were performed using R v2.15.1 [27]. Only female mosquitoes were included in data analysis as they are responsible for disease transmission. Sporozoite rate calculations were based on the total *Anopheles* female catch from CDC light traps and PSC collections. The mean biting rates were corrected with the numbers of sleepers in the houses. Sporozoite rate was estimated, and the annual entomological inoculation rate (EIR) was calculated by multiplying the sporozoite rate by the mean biting rate/night multiplied by 365 days. Due to political instability in the country between December 2007 and March 2008, we were unable to work in our study sites for the months of January, February, and March 2008; hence, data for adult mosquito sampling and malaria incidence for this period are not available in some sites. 

For entomological survey data, the proportion of mosquitoes caught were compared using Chi-square test, while for the other datasets, we perform regression analysis to adjust for the confounding effect of covariates. We fit a negative binomial generalized additive model (GAM) with log link to study the association between risk of malaria parasite and the site while adjusting for body temperature and month, with site entering the model parametrically and month, temperature, and their interaction as nonparametrically smoothed functions. Risk ratios (RRs) were computed for each site in comparison to Nyalenda. Month was defined as a fraction of time given by year + (month 1)/12. To study the association between the risk of having malaria parasite and weather variables, we fit Poisson GAM (with log link) with site, mean monthly relative humidity, monthly rainfall, and temperature as covariates, with the latter two entering the model as smoothed functions and total monthly slides read as the offset. The GAM was used to allow the inclusion of nonparametric smooth functions to model the potential nonlinear dependence of malaria parasites on weather variables and other covariates [28]. The assumption of this model is that

(1) log⁡⁡[E(Y)]=β0+β1x1+β2x2+⋯+Sp−k(xp−k) +⋯+Sp(xp),

where *Y*,is the count of parasites, *E*(*Y*) is the expected value of this count, *X*_*i*_  (*i* = 1,2,…, *p*) are the covariates, and *S*_*p*−*k*_ ⋯ *S*_*p*_ and the smoothing functions.

Negative binomial was assumed for the parametric part in the former case because the observed data were highly aggregated; they had a variance-mean ratio much greater than 1, thus violating Poisson assumptions. The contribution of variables in the regression models was assessed with the use of likelihood ratio (LR) and Wald tests, with all tests performed at 5% level. 

## 3. Results 

**Figure 1:**
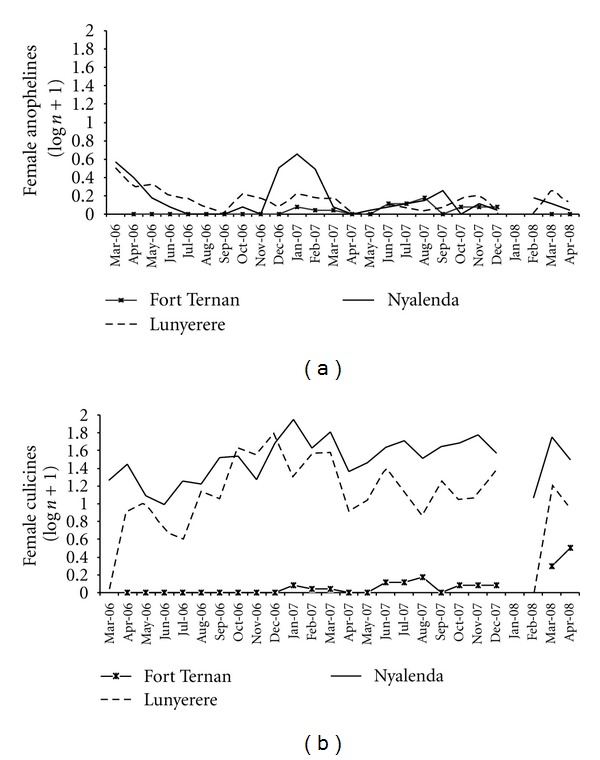
Monthly anopheline (a) and culicine (b) mosquito densities in Fort Ternan, Lunyerere, and Nyalenda.

Of 13640 adult mosquitoes caught, 422 (122 males and 300 females) were anophelines and 13218 (3407 males and 9811 females) culicines.  Figure 1 shows the monthly dynamics of anophelines and culicine mosquitoes collected over time. Of the 300 female *anophelines,* 21, 117, and 162 were collected from Fort Ternan, Lunyerere, and Nyalenda, respectively. Among the 300, a sample of 273 *Anopheles gambiae* was subjected to PCR analysis. The results indicated that overall 85 (31%: 95% CI 26–37%) of the 273 mosquitoes were *An. gambiae. s.s*, but the proportion was significantly greater in Lunyerere (52%) than Nyalenda (19%) and Fort Ternan (5%; *P* < 0.001). For *An. arabiensis, *the overall was 76 (28%: 95% CI 23–33), but the proportion was significantly higher in Nyalenda (50%) than in Fort Ternan (5%) and Lunyerere (3%; *P* < 0.001). For *An. funestus,* the total was 17 (6%: 95% CI 3–9%), detected in two sites only Fort Ternan (68%) and Lunyerere (2%; *P* < 0.001). Other *Anopheles *spp. identified include *An. christyi* and *An. garnhami *(found in Fort Ternan only) and *An. coustani* which was present in all the three sites. Culicine species collected include *Culex* spp., *Mansonia* spp., and* Coquillettidia* spp. In terms of the methods used, the results indicated that overall, when all the female anophelines per house in all the study sites were combined, the CDC light trap (52.3%) caught more mosquitoes compared to PSC (47.7%), although not statistically significant (*P* = 0.689). The mean number of mosquitoes per house per night collected with the PSC was 0.26 ± 0.039, while that of the CDC light trap was 0.15 ± 0.027.

In Fort Ternan, none of the adult anophelines tested by ELISA were found to be infected by *Plasmodium falciparum* sporozoites. However, in Lunyerere, mosquitoes infected with *P. falciparum* were recorded on four occasions in June, September and November in 2007 and March 2008. In Nyalenda, sporozoite-positive mosquitoes were recorded on two occasions in March 2006 and January 2007.

### 3.1. Malaria Sporozoite Rates and the Entomological Inoculation Rate

The mean biting rate was 0.001579, 0.015909, and 0.015238 per person per night in Fort Ternan, Nyalenda, and Lunyerere, respectively. In Fort Ternan, none of the adult* Anopheles* tested by ELISA were found to be infected by *Plasmodium falciparum* sporozoites. Conversely, in Lunyerere and Nyalenda, the *P. falciparum* sporozoite rates were 11.1% and 1.2%, respectively. The entomological inoculation rates per annum were 0.00, 6.91, and 61.79 infective bites/person/year for Fort Ternan, Nyalenda, and Lunyerere, respectively.

### 3.2. Parasitological Impact on Children Cohort Survey

A total of 1735 from Fort Ternan, 1847 from Lunyerere, and 1196 from Nyalenda blood slides were read, of which 60 (3.9%), 106 (6.6%), and 46 (4.2%) slides, respectively, were positive for *P. falciparum, *the only malaria parasite identified from the study population. The mean body temperature was 36.93°C ± 0.01. The proportion of children with malaria parasites was 4.4% over the whole sampling period with significant differences among the study sites (*P* = 0.002). The negative binomial GAM results indicated a significant interaction between temperature and month (*P* < 0.0001). Site, month, and body temperature were all associated with the risk of having malaria parasite (*P* < 0.0001). Compared to Nyalenda, the risk of having malaria parasites was significantly lower in Fort Ternan (RR = 0.48, 95% CI: 0.47–0.49) but significantly higher in Lunyerere (RR = 3.82, 95% CI: 3.45–4.24), after adjusting for body temperature and month.

### 3.3. Impact of Weather Variables on Malaria Transmission

**Figure 2:**
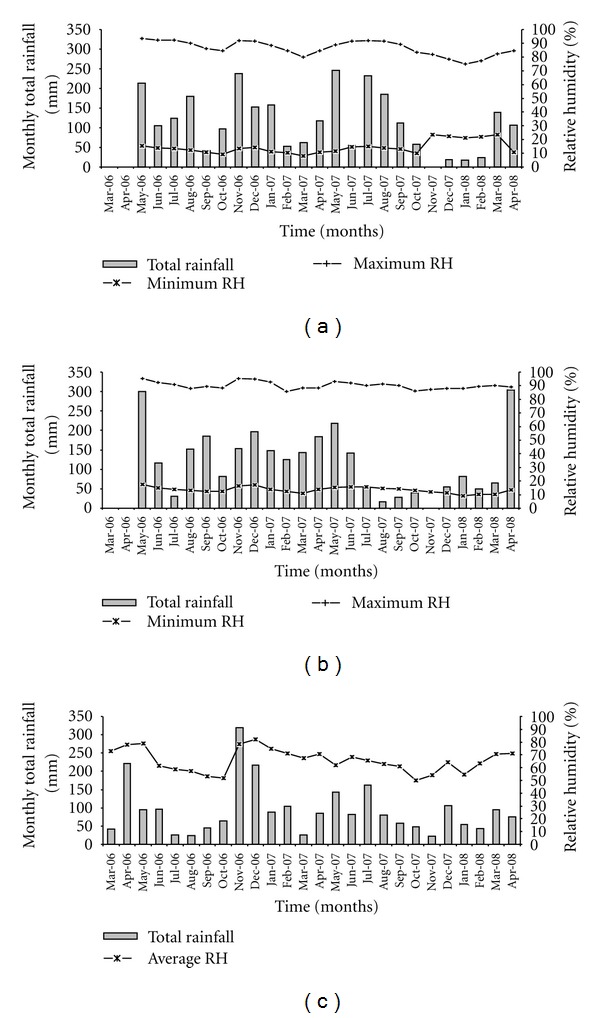
Monthly total rainfall, maximum and minimum or average relative humidity from March 2006 to April 2008, for (a) Fort Ternan, (b) Lunyerere, and (c) Nyalenda.

Overall, the mean monthly rainfall, relative humidity, and temperature were 105.92 ± 10.82, 67.16 ± 1.04, and 20.99 ± 0.26, respectively. A summary of the means (together with their associated standard errors) of the weather variables for each site separately and all sites combined is presented in Table 1, while the monthly rainfall and humidity in Figure 2. Table 1 results show a significantly higher air temperature in Nyalenda than the other two sites. The multivariable Poisson GAM results indicated that rainfall was significantly associated with the occurrence of *P. falciparum* malaria parasites (*P* = 0.0146), but not relative humidity (*P* = 0.2875) and inconclusive for air temperature (LR test *P* = 0.0678) after adjusting for site. The model was able to explain 38.3% of the variance in the data.

## 4. Discussion 

Results of this study show a heterogeneous distribution of vectors and the risk of being infected with malaria within sites only a few kilometres apart. Lunyerere and Fort Ternan, both highland villages at similar altitudes, exhibit markedly different mosquito vector densities and risk of malaria infection. Among the vectors collected, *An. gambiae* s.s. was most abundant in Lunyerere, while *An. arabiensis* was abundant in Nyalenda, whereas *An. funestus* was present in Fort Ternan and Lunyerere only. *Plasmodium falciparum* parasites were recorded among children in all the three sites. Rainfall, body temperature, site, and month were all associated with the occurrence of *Plasmodium* parasites among the children cohort. The differences in malaria risk among the sites can be explained by vector species of local importance, availability of breeding habitats, topography, farming activities [13], terrain characteristic [29], preferred host, and environmental conditions among others. 

*Anopheles gambiae* s.s., the most efficient malaria vector, was abundant in Lunyerere, and due to its anthropophilic behavior, high sporozoite rates were recorded in this site compared to Nyalenda and Fort Ternan. In the same location, the proportion (12.5%) of *An. arabiensis* recorded during the study was high in the Kakamega area when compared to previous studies carried out in nearby villages that did not record the presence of this species [1, 5]. The absence of *An. arabiensis* in Kakamega area at that time was attributed to unfavourable environmental conditions. It therefore follows that the presence of *An. arabiensis* in aquatic habitats [13] and indoors during adult collections indicates that changes in environmental conditions must have occurred to favour its breeding in Lunyerere. A substantial number of *An. funestus* was collected from Fort Ternan, but none was found to be infected with parasites. One possibility is that the species collected might have been a nonvector sibling of the *An. funestus* species complex [23, 30]. We did not carry out PCR analysis to identify the specific sibling species within the *An. funestus* complex. Abundant numbers of *An. arabiensis *and* An. gambiae* larvae were sampled in man-made habitats [13] at the same period in Fort Ternan, but few adult vectors were collected from indoors. Previous work by Koenraadt and others [31] in the same village found few adult anophelines in indoor collections, although anopheline larvae were found in nearby watering sites for cattle and in tyre tracks. The low numbers of vector species caught resting indoors can not explain the presence of malaria parasites in the children cohort. These findings are consistent with the results of Ototo and others [32], who detected no sporozoites in Fort Ternan after eight months sampling, implying that after taking a blood meal the vectors either rest outdoors or perhaps most of the biting takes place outdoors. The results obtained from Lunyerere and Fort Ternan are similar to the findings of Atieli and others [29] who found flat-bottomed valleys to have higher larval and adult densities compared to narrow valleys. In contrast to the highland villages, in the periurban Nyalenda, *An. arabiensis* was the predominant malaria vector compared to *An. gambiae s.s.* both as larvae [13] and adult. Both species contributed equally to malaria transmission. *Anopheles arabiensis* survives better under drier conditions in lowlands than *An. gambiae s.s* [33, 34], and it has been found to dominate irrigated areas such as rice fields [35] among other habitats, which may explain its abundance in Nyalenda. *Anopheles arabiensis* exhibits exophilic and exophagic behavior, and because adult mosquito collections were done indoors throughout this study, we may have missed *P. falciparum *sporozoite-positive mosquitoes of this species in all sites. Recent studies suggest existence of outdoor biting malaria vectors that may contribute to considerable transmission, which went hitherto unnoticed [36, 37]. It would therefore be beneficial for future studies to consider incorporating both indoor and outdoor mosquito catches. *Anopheles coustani,* a potential vector species for malaria transmission [38], was present in all sites.

The risk of being infected with malaria was lower in Fort Ternan but higher in Lunyerere when compared to Nyalenda. These findings can partly be explained by the annual EIRs recorded which show distinct differences among the study sites with the rural village of Lunyerere having a higher EIR (61.79) in contrast to Fort Ternan where the EIR was zero throughout the study period. The periurban Nyalenda recorded an EIR (6.91) eleven times lower than that of Lunyerere. The differences in farming activities can also explain the vast differences between the sites. For Lunyerere, small-scale food crop production in a transformed swamp area with underground water seepage ensures vector breeding throughout the year which is not the case with Fort Ternan, an area under large-scale farming. Nyalenda being relatively flat, transformed swamp area supplied with water throughout the year with a significantly higher average air temperature compared to the other two, one would expect a higher risk to infection here. The presence of breeding habitats throughout the year with favourable temperatures leads to accelerated sporogonic cycle of *P. falciparum* parasites hence posing a higher risk of infection but that was not the case when compared to Lunyerere. Nyalenda being a periurban site, the population was found to be equipped with more knowledge on mosquito and malaria control than the counterparts in the two villages. Consequently, more households used protective measures such as insecticide-treated bednets [39] lowering the risk of being infected. Finally, microscopy was adopted as the standard method used to examine malaria parasites in a cohort of children throughout the study period. Although microscopy still remains the standard diagnostic method for malaria parasites, a number of studies have shown that it may fail to detect low parasitemia levels that are common in asymptomatic individuals when compared to PCR [40, 41]. Asymptomatic individuals are able to sustain malaria transmission [40], and thus, the failure of microscopy to identify such individuals could mean that our result represents an underestimate of the real situation. Nevertheless, historical records of malaria parasite prevalence in the highlands of Western Kenya reported higher rates than what was found in the current study [42]. The reduction in malaria cases is attributed to the adoption of the Roll Back Malaria initiative [43] by the Kenyan government since the year 2006, which scaled up the use of insecticide-treated bednets [ITNs] in the areas studies [44]. However, in spite of these control measures, low levels of transmission continue given the recorded *P. falciparum* parasite, calling for integrated approaches that are complementary to the use of ITNs.

In conclusion, these results show that the risk of being infected with malaria in Western Kenya is heterogeneously distributed, both temporally and spatially depending on the topography, farming activities such as swamp reclamation, biology of vector species concerned, availability of mosquito breeding habitats, and behavioural characteristics of the population at risk. The presence of malaria parasite-infected children in all the study sites provides evidence of local malaria transmission, although in Fort Ternan the mosquito density was too low to explain the presence of malaria parasites in the cohort of children. Future studies need to consider both indoors and outdoor resting vectors to get a more insight on how malaria is transmitted in Fort Ternan.

## Figures and Tables

**Table 1:** Means (standard errors) for the weather variables for each site separately and all sites combined.

Weather variable	Site								
	All sites		Nyalenda		Fort Ternan		Lunyerere		*P* value
	Mean	se	Mean	se	Mean	se	Mean	se
Rainfall	105.92	10.82	78.98	11.52	122.22	18.89	112.31	22.35	0.2477
Relative humidity	67.16	1.04	64.78	1.93	66.46	2.08	69.91	1.13	0.1229
Air temperature	20.99	0.26	23.14	0.19	19.94	0.30	20.17	0.24	<0.0001*

*Mean air temperature was significantly different among the three sites, but not rainfall and relative humidity.
